# Synthesis of Imine-Naphthol Tripodal Ligand and Study of Its Coordination Behaviour towards Fe(III), Al(III), and Cr(III) Metal Ions

**DOI:** 10.1155/2014/915457

**Published:** 2014-09-08

**Authors:** Kirandeep Kaur, Minati Baral

**Affiliations:** Department of Chemistry, National Institute of Technology, Kurukshetra, Haryana 136119, India

## Abstract

A hexadentate Schiff base tripodal ligand is synthesized by the condensation of tris (2-aminoethyl) amine with 2-hydroxy-1-naphthaldehyde and characterized by various spectroscopic techniques like UV-VIS, IR, NMR, MASS, and elemental analysis. The solution studies by potentiometric and spectrophotometric methods are done at 25 ± 1°C, *µ* = 0.1 M KCl, to calculate the protonation constants of the ligand and formation constants of metal complexes formed by the ligand with Fe(III), Al(III), and Cr(III) metal ions. The affinity of the ligand towards Fe(III) is compared with deferiprone (a drug applied for iron intoxication) and transferrin (the main Fe(III) binding protein in plasma). Structural analysis of the ligand and the metal complexes was done using semiempirical PM6 method. Electronic and IR spectra are calculated by semiempirical methods and compared with experimental one.

## 1. Introduction

Studies of multidentate ligands have experienced a tremendous upsurge because such ligands mimic the environment of metal ions in biological systems [1]. These ligands provide various coordinating sites to the guest metal ions, which can fulfill the primary valency and result in novel structural motifs. Multidentate ligands can have cyclic, linear, branched, and tripodal nature [2]. Cyclic ligands offer high selectivity but slower metalation kinetics. Linear and branched ligands show faster metalation kinetics but less selectivity and easy demetalation. The tripods show the advantages of both types.

Due to such advantages and, also, for the ease of formation, work has been done on the synthesis of tripodal ligand(s), especially the Schiff base tripodal ligand(s). Moreover, the tren based Schiff bases have drawn special interest as they offer a number of applications: chemosensing and fluorosensing, in chelation therapy for the treatment of metal overload, clinical diagnosis, and chemotherapy [3–5]. The metal complexation of such ligands has been extensively studied with trivalent metal ions such as Fe(III), Cr(III), and Al(III) and is found to form uncharged complexes with high thermodynamic stability and kinetic inertness [6–13]. The coordination chemistry with Fe(III) with tripod systems has been explored with respect to their potential application in iron overload treatment [14–17]. The search to develop new effective chelators for the treatment of aluminium intoxication [18, 19] leads to the complexation study of Al(III) with tripodal chelators. Removal of Cr(III) is also extremely necessary as it leads to formation of Cr(VI), a highly toxic form and known to have mutagenic and carcinogenic properties [20, 21]. Such tripodal chelators are known to form strong complexes with Cr(III) and hence can be explored for Cr(III) removal.

**Figure 1:**
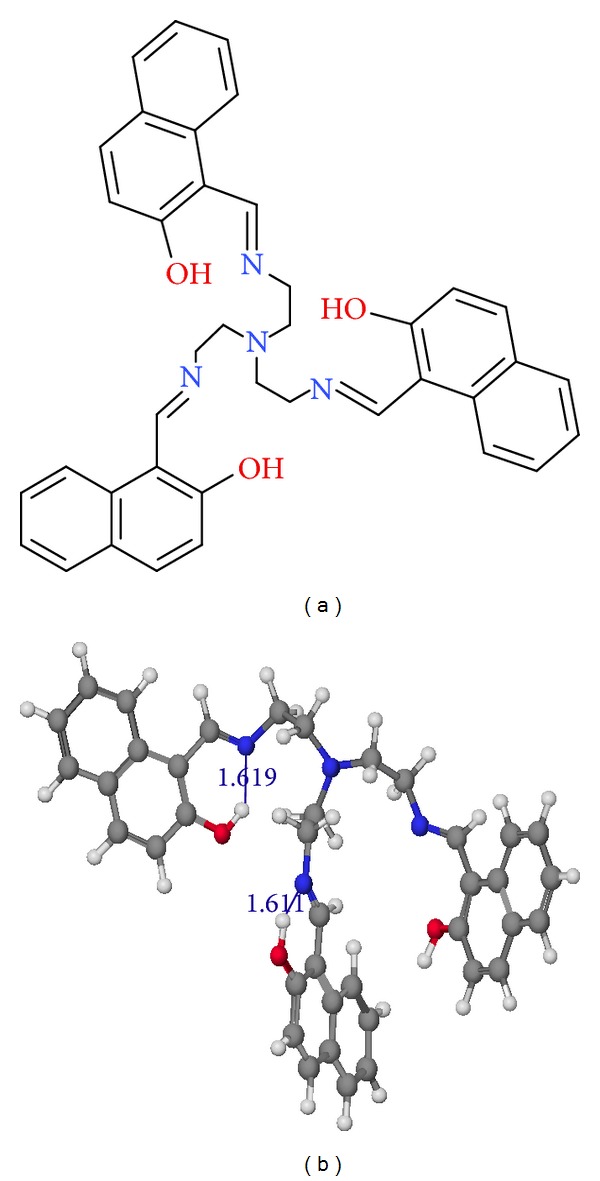
(a) Molecular structure of trenhynaph; (b) least strain energy structure of ligand obtained from semiempirical PM6 method showing presence of hydrogen bonding.

Considering the extensive usage of tripodal multidentate chelating systems, we in our present work report the synthesis of a novel multidentate imine-naphthol based tripodal ligand along with spectroscopic details and discussion of keto-enol tautomerism shown by the ligand (Figure 1). The coordination behaviour of the ligand with the three trivalent metal ions Fe(III), Cr(III), and Al(III) studied by potentiometric and spectrophotometric methods is also reported. The protonation constants of the ligand and formation constants of the metal complexes are determined. Some theoretical studies carried out to shed light on the structural elucidation and binding modes in the complexes by using semiempirical method are also reported.

## 2. Experimental

### 2.1. Materials and Measurements

All chemicals and solvents were of analytical grade available commercially. Potassium hydroxide and hydrochloric acid were obtained from Fisher Scientific. 2-Hydroxynaphthaldehyde and Tris (2-amino ethyl) amine (tren) were obtained from Sigma Aldrich. Anhydrous ferric chloride, aluminium sulphate, and chromium chloride were procured from Qualigens Fine Chemicals (Fisher Scientific). Tetrahydrofuran and dimethyl sulfoxide were purchased from Merck. Chemicals were used as purchased without purification. Solvents were dried using standard methods [22].

Melting points (MP) were determined on a microsil (India) MP apparatus and are uncorrected. They are given in degree centigrade (°C). Electronic spectra were taken on Hitachi spectrophotometer U 0080D. Infrared (IR) spectra were recorded on a Perkin-Elmer RX 1 FT-IR spectrometer using KBr discs. ^1^H and ^13^C NMR spectra were recorded on a Bruker Avance II 400 NMR spectrometer, at SAIF, Panjab University, Chandigarh, using tetramethylsilane (TMS) as an internal reference. The chemical shifts are mentioned in parts per million (ppm). The mass spectrum was performed on Waters Micromass Q-TOF Micro with electron spray ionization (EI) technique at SAIF, Panjab University, Chandigarh. C, H, N, S, and O analysis was carried out on EURO EA 3000. Potentiometric studies were carried on Sension 02 pH meter.

### 2.2. Synthesis of 1-[(E)-2-[Bis[2-[(2-hydroxy-1-naphthyl)methyleneamino]ethyl]amino]ethyl iminomethyl]naphthalen-2-ol (trenhynaph)

In a round bottom flask 10 mL of THF was taken, added to it 3 g (0.0174 mol) of 2-hydroxynaphthaldehyde, and the contents were stirred till complete dissolution. A solution of 0.0846 g of tren in 10 mL of THF was added dropwise. Solution was allowed to stir for 1 hr. Very thick and bright yellow coloured precipitates were obtained, which were filtered, washed with diethyl ether, and dried in vacuum. Yield (2.28 g, 76%), MP: (185–189)°C, colour (yellow), IR (KBr pellet, cm^−1^): 3055 (Ar=C–H), 2940(–CH_2_, asymmetric), 2821(–CH_2_, symmetric), 1622(–C=N), 1350(–C–O), 764(=C–H, OOP); ^1^H NMR (DMSO-D_6_, *δ* ppm) 3.00 (t, *J* = 12.48, 6H, H-12), 3.75 (t, *J* = 4.64, 6H, H-13), 6.68 (d, *J* = 9.32, 3H, H = 3), 7.13 (d, *J* = 14.6, 3H, H = 6), 7.32 (t, *J* = 14.9, 3H, H = 7), 7.53 (d, *J* = 7.72, 3H, H = 5), 7.60 (d, *J* = 9.16, 3H, H = 4), 7.98 (t, *J* = 8.82, 3H, H = 8), 9.04 (d, *J* = 9.76, 3H, H = 11), 14.11 (d, *J* = 3.84, 3H, H = 14); ^13^C NMR (DMSO-D_6_, *δ* ppm) 177.26, 159.05, 136.76, 134.14, 128.58, 127.53, 125.07, 125.33, 121.87, 118.58, 105.77, 38.93, 40.18; mass spectrum (ESI) molecular ion [M+2] peak *m*/*z* = 610.4, [M+1] peak *m*/*z* = 609.3; anal. calcd. for C_39_H_36_O_3_N_4 _(608.27); C,77; H,9; N,9.2; O,7.9; found: C,77.23; H,9.36; N,9.35; O,7.98.

### 2.3. Titration Procedure

Potentiometric titrations were carried out in order to find out the protonation constant of the ligand and formation constant of the metal complexes. The temperature for the experiment was maintained at 25 ± 1°C. A titration cell which has double glass walls was used and attached to circulatory bath to maintain the consistency of temperature. Sension 02 pH meter in combination with glass electrode was used to measure the pH. The whole equipment set was calibrated according to the standard methods [23].

Doubly distilled deoxygenated and deionized water was used to prepare all the solutions used in the experiment. KOH solution (0.1 M) was prepared in 5 : 95 ratio of DMSO/water. HCl solution (0.1 M) was prepared in water. Ionic strength of the solution was maintained 0.1 M by adding 1 M KCl solution. The solution of ligand of strength 0.01 M was prepared in DMSO and solution of metal 0.01 M in water. The final concentration of ligand and metal in titration cell was maintained at 0.001 M. The ratio of DMSO/water in the resulting titration cell was maintained at 5 : 95 by adding appropriate solutions. Following titrations in which metal to ligand ratios C_M_/C_L_ (0 : 1) and C_M_/C_L_ (1 : 1) were carried out. The data obtained from the experiment is fed into the least square fitting program HYPERQUAD 2006 and manual fitting of experimental and theoretical data was done to obtain the protonation constant of the ligand and the formation constants of metal complexes. The whole titration procedure is carried out twice to confirm the results.

Spectrophotometric titrations were carried out in the same conditions as for potentiometric titrations. In these studies a dilute solution of ligands (1 × 10^−5^ M) and metal ion (1 × 10^−5^ M) was made acidic by adding appropriate amount of 0.1 M HCl at an ionic strength of 0.1 M KCl and titrated against standard 0.1 M KOH solution. After each addition of the base sufficient time was given to attain the equilibrium, when a constant value of pH was attained and then an aliquot of the solution was taken out to take the spectra. Transfer of the solutions was done very carefully to minimize the error due to volume loss. Computer program pHAb was used to determine the protonation constants of ligand and formation constants of metal complexes.

### 2.4. Theoretical Studies

Whole theoretical study was performed on the machine Pentium (R) IV having windows XP environment 3.20 GHz CPU using computational program HYPERCHEM version 7.5 [24] and CACHe work system Pro version 7.5.0.85 [25]. The minimum strain energy of the molecule and the metal complexes was obtained through molecular mechanics method using MM^+^ force field. The initial structure obtained was reoptimized by semiempirical method using PM6 self-consistent field (SCF) at Restricted Hartree-Fock Polak-Ribiere method. Semiempirical ZINDO/1 method was applied to calculate the vibrational spectra. ZINDO using INDO/S parameters is used for calculating electronic spectra after reoptimizing geometry with MOPAC PM5.

## 3. Results and Discussions

### 3.1. Synthesis and Characterization

**Figure 2:**
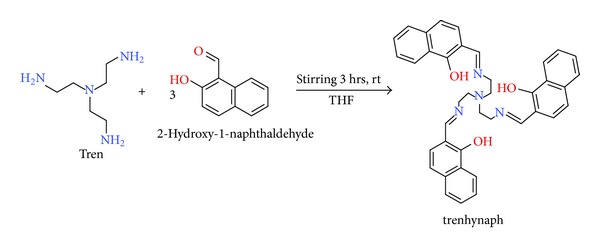
Scheme for the synthesis of trenhynaph.

The ligand trenhynaph was synthesized by condensation of triamine (tren) and aldehyde (2-hydroxynaphthaldehyde) as per the scheme shown in the Figure 2. It is bright yellow in colour and found to be soluble completely in DMSO and DMSO/water ratio above 5 : 95. It is also partially soluble in methanol and ethanol but insoluble in other solvents like chloroform, ether, acetonitrile, acetone, and so forth.

**Scheme 1:**
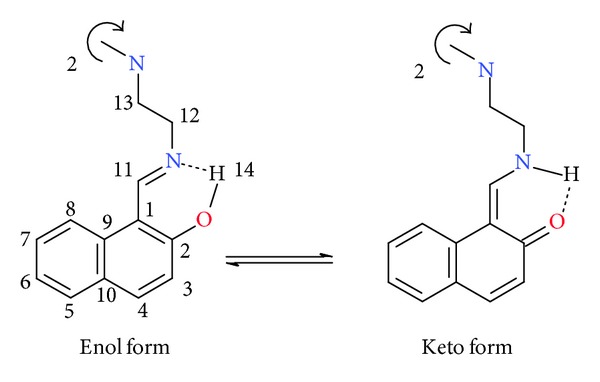
Keto-enol forms of trenhynaph.

The ligand was characterised on the basis of elemental analysis, UV-VIS, IR, ^1^H NMR, ^13^C NMR, and MASS spectra. It has been studied earlier that the presence of ortho hydroxyl group in Schiff bases favours the existence of intramolecular hydrogen bonds and the tautomerism, which accounts for the formation of either phenol-amine or keto-amine tautomer (Scheme 1). For the Schiff bases of salicylaldehyde with amines (–NH) form is found to be less stable. The salicylaldimines exist predominantly in –OH tautomeric form in crystalline state and the reason behind this is the loss of ring aromaticity [26]. But 2-hydroxynaphthylidenes derivatives show the presence of both the tautomers, i.e., keto-amine and enolimine. In naphthalimides (–NH) form is expected to be more stable due to resonance and delocalization in the retained aromatic structure [27, 28].

**Figure 3:**
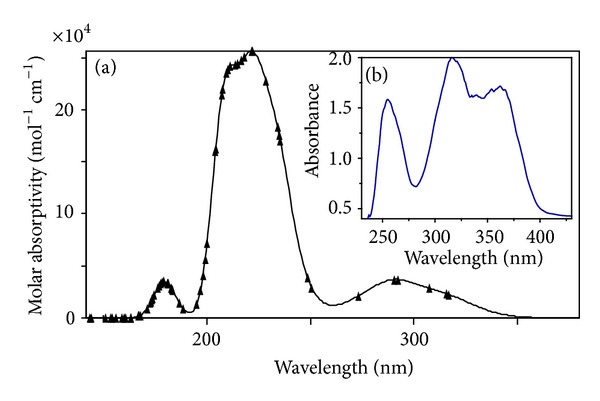
(a) Theoretical electronic spectra of ligand trenhynaph by ZINDO using INDO/S parameters after reoptimizing geometry with MOPAC PM5 and (b) experimental electronic spectra of the ligand (1 × 10^−5^ M in DMSO/H_2_O, 5/95).

Matijević-Sosa et al. investigated a number of 2-hydroxy-1-naphthaldehyde Schiff bases through various spectroscopic techniques like IR, one and two dimensional, homo-heteronuclear ^1^H, and ^13^C NMR. It was established that in solution (DMSO) the (–NH) tautomer predominates while the IR spectra of solid samples (KBr) suggest that the Schiff bases exist in solid state as (–OH) isomer [29, 30]. Considering these previous observations we investigated the spectra of our ligand and found that the above same trend is being followed in our case also. IR spectra suggest the predominance of the (–OH) isomer and ^1^H and ^13^C NMR (DMSO-d_6_) suggest the (–NH) tautomer. Electronic spectrum of trenhynaph exhibits three peaks. Peaks obtained at 254.9 nm and 315.99 nm can be attributed to the *π* → π* transitions due to naphthyl ring and 365.99 nm *n* → π* transitions associated with the imine bond, Figure 3(b). Similarly the calculated electronic spectra also give three peaks, Figure 3(a). Although the calculated electronic signals are not the same as the theoretical one, still they follow the same trend as that of the experimental one.

IR spectra show a strong band at 1622 cm^−1^ which can be attributed to *ν* (–C=N) and they confirm the condensation of amine and aldehyde. The sharp peak obtained at 1350 cm^−1^ which can be due to the *ν* (–C–O) suggests in solid state that it exists as –OH tautomer. Aromatic =C–H stretching is obtained at 3055 cm^−1^ and =C–H out of plain bending is obtained at 764 cm^−1^. The low intensity peaks obtained at 2940 cm^−1^ and 2821 cm^−1^ can be assigned to asymmetric stretching vibrations and symmetric stretching vibrations of the methylene (–CH_2_) group of parent tren moiety. The IR spectrum of the ligand is also obtained theoretically by using semiempirical ZINDO/1 method. The theoretical values were not in quantitative agreement with the experimental values but the trend of the peaks follows the same pattern as in Table 1.

Skeletal structure of the ligand is revealed by NMR spectra of the ligand. The ^1^H NMR spectra show peaks at 3.0039 ppm (t, 6H) and 3.7633 ppm (t, 6H) which can be attributed to the methylene protons shown in Scheme 1 by numbers 12 and 13, respectively. The signal obtained at 9.0615 ppm (d, 3H) shows the presence of imine proton number 11. The peak obtained at 6.698 (d, 3H), 7.6177 (d, 3H), 7.547 (d, 3H), 7.1334 (t, 3H), 7.3272 (t, 3H), and 7.9926 (d, 3H) corresponds to the proton numbers 3, 4, 5, 6, 7, and 8, respectively, as shown Scheme 1. A peak at 14.1123 ppm (d, 3H) is also obtained corresponding to the proton of imine nitrogen when ligand exists in keto-imine form. Similarly ^13^C NMR spectra also show the presence of signal at 177.26 ppm which corresponds to the carbonyl carbon C2. The signals obtained at 105.77, 121.87, 136.76, 134.14, 125.07, 128.58, 118.31, 127.53, and 125.33 correspond to the carbon atom numbers 1, 3, 4, 5, 6, 7, 8, 9, and 10, respectively. The signal at 159.05 ppm corresponds to carbon atom number 11. Peaks for methylene carbon atoms are obtained at 38.93 and 40.18.

Mass spectrum of the ligand shows molecular ion peak at *m*/*z* 609.3, which was expected at 608.27. The base peak with 100% intensity is obtained at *m*/*z* 455.2 which corresponds to the fragment C_28_H_30_N_4_O_2 _(454.2). One more important peak at *m*/*z* 281.1 corresponds to the fragment C_13_H_23_N_3_.

### 3.2. Ligand Protonation Constant

**Figure 4:**
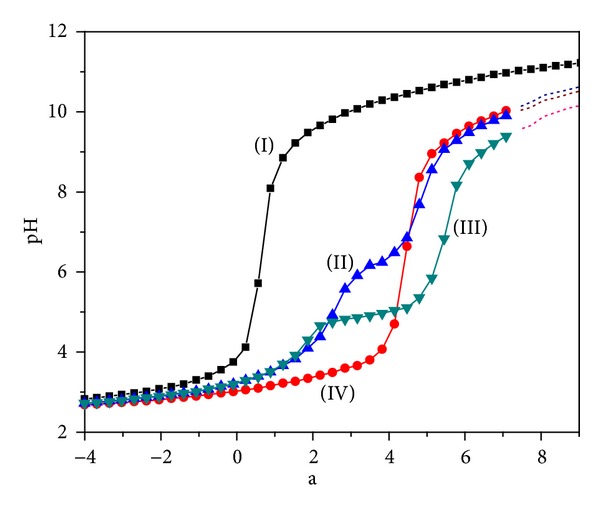
Potentiometric titration curves: (I) 1 × 10^−3^ M trenhynaph (L), 1 × 10^−3^ M [trenhynaph][M(III)] (1 : 1 molar ratio) [(II)-Al, (III)-Cr, (IV)-Fe], *T* = 25 ± 1°C, 0.1 M KCl, and “*a*” is the moles of base added per mole of ligand/complex.

The formation of metal complexes is viewed as competition between the protons of ligand moiety and metal ions; hence, protonation constants were evaluated experimentally, which are required to be used as input data to calculate the formation constants of the metal complexes. Potentiometric studies to investigate the protonation constants were carried out in DMSO/water (5 : 95) mixture at *µ* = 0.1 M KCl and 25 ± 1°C. The potentiometric curves between pH and “*a*” where “*a*” is the moles of base added per mole of ligand present are shown in Figure 4. Up to the volume of *a* = 0 only the excess of acid is being consumed. After *a* = 0 mL drastic increase in pH is observed and this indicates the deprotonation of the ligand. Inflection point at *a* = 3 indicates the release of three moles of protons from the ligand.

The potentiometric data on quantitative analysis by using the software Hyperquad 2006 [31] gave the acceptable best fit for the three protonation constants for the tripod. The following are the proposed reactions which are being followed by the ligand during its protonation process:

(1) LH⁡n−1+H⇌LHn,  Kn  =  [LH⁡n][LH⁡n−1][H],

where  L(ligand), *n* = 1,2, 3.

If we examine our ligand structure, it is found that the tripodal ligand(s) in its fully protonated form have seven protons which can be deprotonated by the addition of base. These seven protons include three protons of tertiary imine nitrogen and three protons of naphtholic oxygen and one proton associated with tertiary amine of tren capping. But under the present investigation only three protonation constants can be calculated, which corresponds to the protons of naphtholic oxygen Table 2. The protonation constant for the three protons of imine nitrogen cannot be calculated because when an aromatic alcohol system forms a Schiff base, the imine nitrogen possesses very low basicity with log⁡⁡K_a_ of 1.53 and 2.84 [32]. It is found to be so due to the –I effect of the substituted aromatic naphthol group which reduces the electron density of imine nitrogen. The apical nitrogen atom is also very acidic with  pK_a_ < 1.5 [8]. So, the ligand is considered to be triprotic LH_3_ and the calculated protonation constants correspond to the naphtholic protons. The protonation constant values are found to be lower than naphthol; it may be caused by the intramolecular hydrogen bonding supported by the least strain energy structure of the ligand (Figure 1).

**Figure 5:**
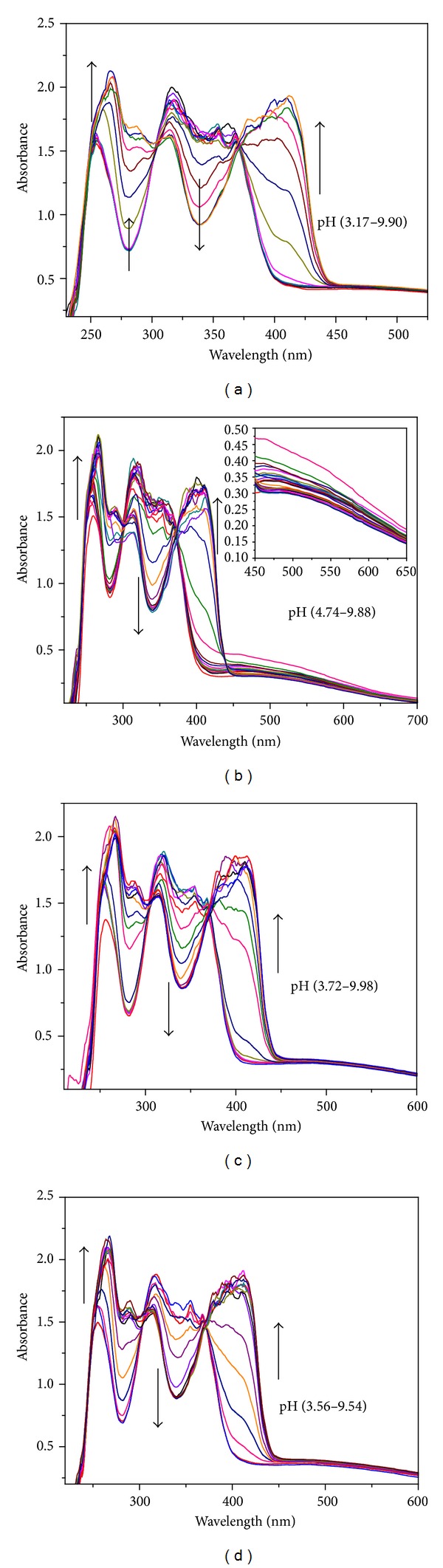
pH dependent electronic spectra as a function of absorbance and wavelength for (a) trenhynaph, (b) 1 : 1 solution of Fe(III), (c) Cr(III), and (d) Al(III) and trenhynaph, [trenhynaph] = [M(III)] = 1 × 10^−5^ M, *T* = 25 ± 1°C, 0.1 M KCl in DMSO/H_2_O (5 : 95).

Protonation constants of the ligand were also calculated by spectrophotometric method. In this method a pH versus absorbance measurement was carried out at ligand concentration (1 × 10^−5^ M), 25 ± 1°C, and constant ionic strength 0.1 M KCl. The absorbance spectra of the ligand were recorded within the pH range (3.17–9.90) and are shown in Figure 5(a). No appreciable changes in the electronic spectra were observed below the experimental pH 3.17 and above pH 9.90 and spectra overlapped with each other. The state of equilibrium between various protonated and deprotonated species was examined from the spectral changes like shifting of peaks and formation of isosbestic points. The whole range of spectral data as shown in the Figure 5(a) was included in the calculations by nonlinear least square fitting program pHAb, which gave best fit for three species whose protonation constants agreed well with the potentiometric results.

The solution remained yellow in the entire pH range. The ligand exhibited three peaks with maxima at 254.9 nm; 315.99 nm can be attributed to the *π* → π* transitions due to naphthyl ring and 365.99 nm *n* → π* transitions associated with the imine bond. Peak obtained at 254.9 nm showed a bathochromic and hyperchromic shift. A new peak appeared at 275 which showed a concomitant hyperchromic shift. Another peak at 315.99 nm due to *π* → π* transitions associated with naphthyl ring showed lowering of intensity with increasing pH. Absorption band at 365.99 nm experiences a bathochromic and hyperchromic shift. The shifting of the peaks to longer wavelength can be explained by the fact that, on deprotonation, formation of naphtholate ion takes place, which can stabilize the π* excited state due to charge delocalization, bring the lowest excited closer to the highest ground state, and thus permit a lower energy (longer wavelength) for transition. Since the imine bond is in conjugation with the ring the peak at 365.99 nm also experiences red shift.

**Figure 6:**
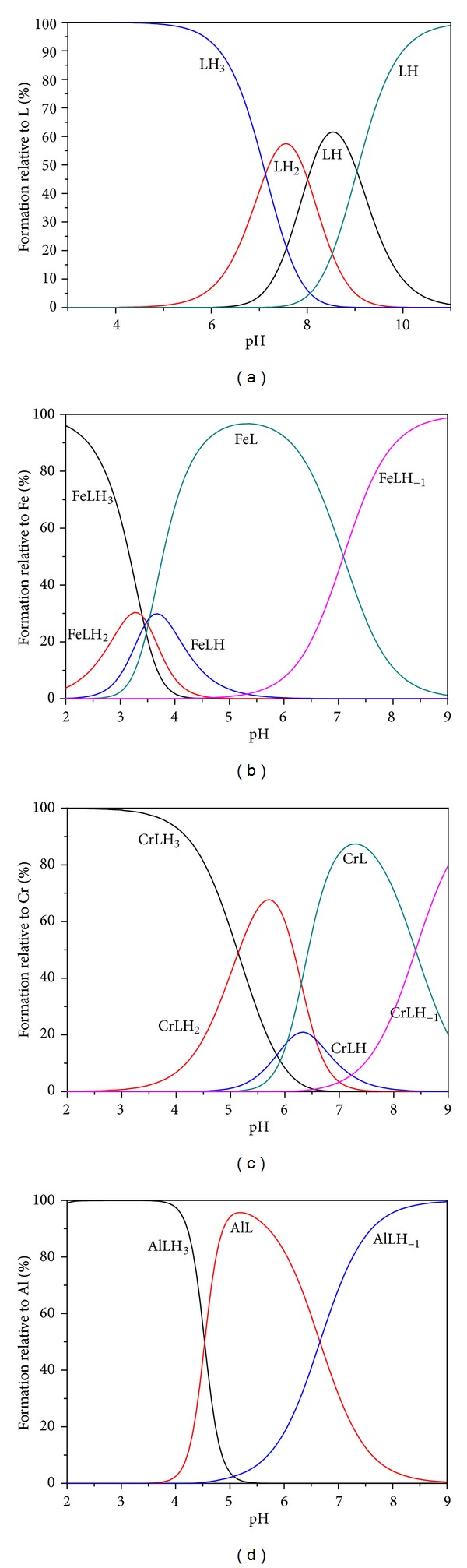
Species distribution curves computed from the (a) protonation constants for trenghynaph and (b) formation constants for [trenhynaph: Fe(III)], (c) Cr(III), and (d) Al(III).

From the diagram, the speciation diagram obtained by using HYSS program [33], it can be inferred that at initial stages the ligand remains 100% in its fully protonated form LH_3_, Figure 6(a). The dominance of the LH_3_ species starts to decrease as the pH increases. With the increase in pH the deprotonation of the ligand starts to form species LH_2_, LH, and L. After pH~5 LH_2_ begins to form and exists up to pH~9, though in very low amount. LH is formed predominantly in the pH range 7–9. Fully deprotonated species of the ligand L shows its formation at pH~7.5 and becomes predominant after pH 9.

### 3.3. Metal Complex Formation

Figure 4 shows the potentiometric titration curves of the ligand in presence of M(III) where M = Fe, Al, and Cr in 1 : 1 metal-ligand ratio. The metal ligand curves are showing considerable changes with respect to the curves of the ligand, which qualitatively implies that the metal complexation has occurred and the shape of the curve also gives an idea about the good affinity of ligand towards metal ions. The potentiometric curve for Fe(III) lies below the curve for Cr(III) and Al(III) indicating better complexation with Fe(III). The break point obtained at the value *a* = 4 corresponds to the release of four moles of protons of the ligand prior to deprotonation from hydroxyl group. Further addition of KOH increases pH and another inflection point is obtained at *a* = 7 indicating overall release of seven protons from the ligands. Above pH~9.5 the solution becomes turbid due to the appearance of precipitate, which may be caused by hydrolysis of the metal complexes. Taking these observations in consideration, a number of models were tried in the program Hyperquad 2006 to calculate the formation constant. The best fit was obtained while considering the species MLH_3_, MLH_2_, MLH, ML, and MLH_−1_ for Fe(III), Cr(III), and MLH_3_ and ML and MLH_−1_ for Al(III) Table 3. The following equations show the equilibrium reactions for the overall formation constants:

(2) M+L+nH=MLHn,  β11n  =  [MLHn][M][L][H]n,

where L(ligand), M(Fe(III), Cr(III), Al(III)), *n* = 0,1, 2,3, −1.

Spectrophotometric method was also employed to calculate the formation constant in order to support potentiometric outcomes. The spectrophotometric titrations were carried out in DMSO/H_2_O (5/95) solution with ligand and metal concentration of 1 × 10^−5^ M at 25 ± 1°C and ionic strength 0.1 M KCl. The electronic spectra of ligand in presence of equimolar metal ions Fe(III), Al(III), and Cr(III) were recorded in the pH range 2–10 and significant spectral changes observed in different pH ranges are given in Figures 5(a), 5(b), and 5(c). Shifting of the ligand peaks was observed along with the formation of isosbestic points with gradual increase of pH. In Fe(III)-trenhynaph metal complex, one of the characteristic absorption bands (*π* → π*) for the naphthyl ring observed at 254.9 nm, experiences a red shift along with the appearance of new low intensity peak located at 277 nm. The other peak with maxima at 315.99 nm was observed with lowering in intensity. The peak associated with *n* → π* transition centered at 365.97 nm also showed a red shift with a hyperchromic effect. Formation of isosbestic points at 280, 300, 370, and 440 nm due to shifting of the peaks indicates the involvement of hydroxyl group of naphthyl ring and the imine nitrogen in complexation. An additional band due to metal ligand charge transfer band is also observed for the Fe(III) complex located at 540 nm inferring a hexacoordinated environment. Similar spectral variations are observed for chromium and aluminium metal ions suggesting similar modes of coordination as for iron.

**Figure 7:**
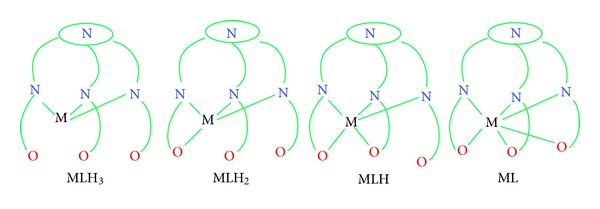
Suggested coordination modes of ligand with Fe(III), Cr(III), and Al(III).

Species distribution curve obtained from the program HYSS using the calculated formation constant values is shown in Figures 6(b), 6(c), and 6(d). It indicates that the formation of the complex occurs at pH~2 for Fe(III), Cr(III), and Al(III). The very first species formed is MLH_3_; further increase in the pH leads to the formation of MLH_2_, MLH, ML, and MLH_−1_ for Fe(III), Cr(III), and ML and MLH_−1_ for Al(III). MLH_3_ type of complex may be formed due to the interaction of weakly basic amine nitrogen atoms. In this complex species the metal ion is face capped by the ligand. The three remaining coordinating sites may be occupied by water molecules to satisfy the primary valency of the metal ion. The Fe(III)L species began to form at pH = 3, while for chromium it appeared after pH = 4 and for aluminium it was formed after pH = 5 which support the better complexation with Fe(III) metal ion. The various probable coordination modes are shown in Figure 7. Addition of more bases will result in the replacement of water molecules by the naphtholic hydroxyl groups to form the subsequent species MLH_2_, MLH, and ML. The formation of the species MLH_−1_ is due to the deprotonation of ML. But there is no deprotonating site in ML. Thus, the formation of MLH_−1_ may possibly be due to the deprotonation of water molecules linked with the imine linkage, resulting in the breaking of Schiff base [34].

**Figure 8:**
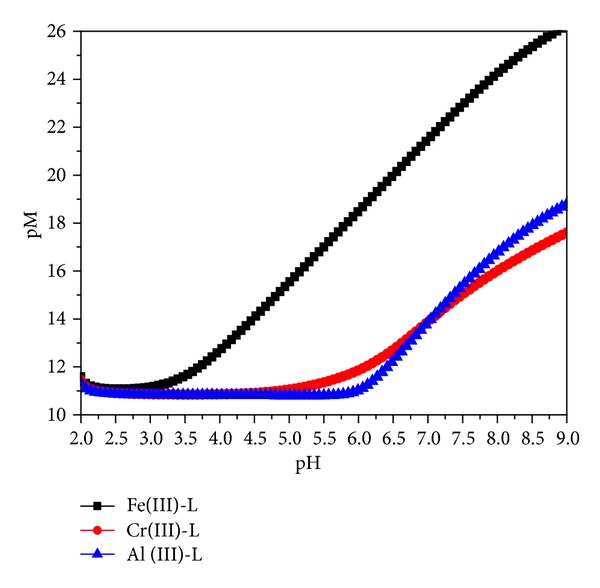
pH versus pM graph: pM was calculated for [L] = 5 × 10^−4^ and [M] = 5 × 10^−5^ using protonation constant of ligands and complexation constants *β*_11*n*_.

Results obtained for the ligand under present investigation are compared with two similar (N_3_O_3_) type ligands* cis*,*cis*-1,3,5-tris[(2-hydroxy-benzylidene)aminomethyl]cyclohexane (L^1^),* cis,cis-*1,3,5-tris[1-(2-hydroxyphenyl) ethylideneaminomethyl]cyclohexane (L^2^), deferiprone (medically applied for the iron intoxication) and transferrin (the main Fe binding protein in plasma). The pFe values for ligands L^1^, L^2^, deferiprone, and transferrin at pH = 7.4 are 20.14 and 16.41, 19.3, and 20.3, respectively [9]. These values are calculated using [L]_total_ = 5 × 10^−4^ M, [Fe(III)]_total_ = 5 × 10^−5^ M, deprotonation constants of the ligand, and the formation constant of metal complexes. The ligand in the current investigation has pM = 21.82 at the same concentration of ligand and metal. This indicates that our ligand has better complexation ability for Fe(III) at pH 7.4 than ligands L^1^, L^2^, deferiprone, and transferrin. So the ligand can be explored for their potential application for iron overload chelation therapy. For the metal ions Fe(III), Al(III), and Cr(III) at pH = 7.4 the pM values are 21.82, 14.93, and 15.28, respectively. The highest value is obtained for Fe(III). The ligand selectively binds with Fe(III) ions at pH 7.4. To check whether this selectivity order is followed for whole pH range graphs between pH and pM drawn Figure 8. Curves show that the selectivity for Fe(III) ions remains greater than the other metals for the whole pH range.

**Figure 9:**
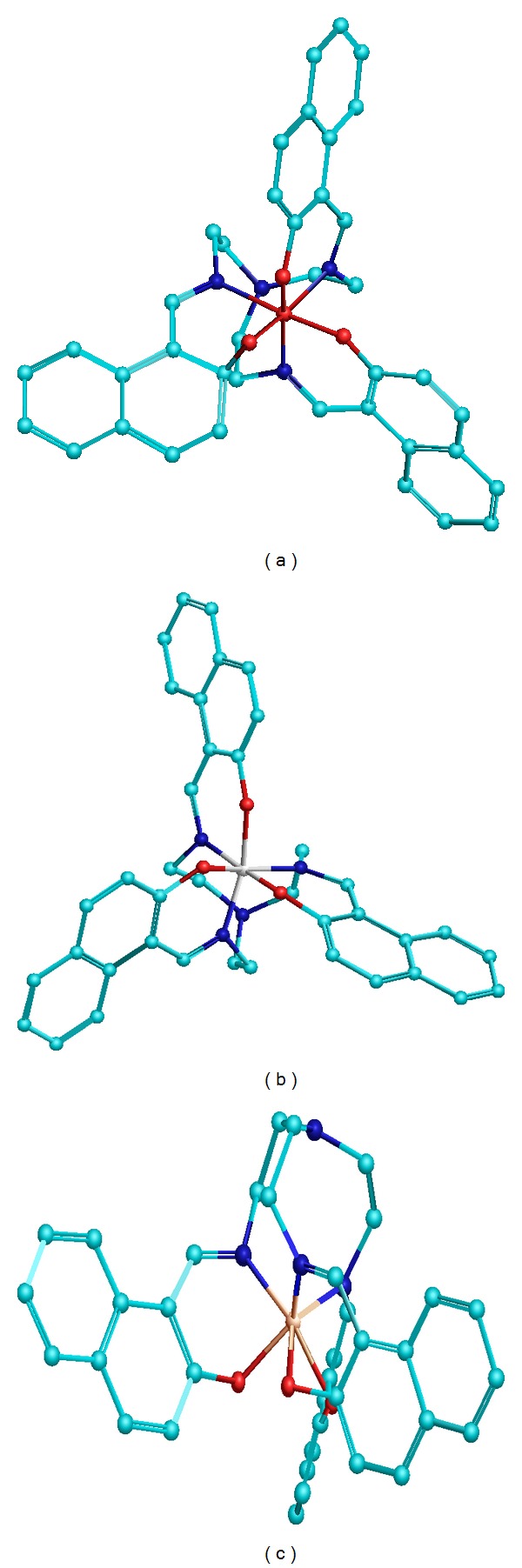
Least strain structures of ML type metal complexes for (a) Fe(III), (b) Cr(III), and (c) Al(III) using semiempirical PM6 method at Hatree-Fock algorithms.

In order to validate the formation of the above said complexes for the three metal ions the structural investigation of all the species formed in solution (MLH_3_, MLH_2_, MLH, ML, and MLH_−1_) was performed using PM6 semiempirical method. The energy value thus obtained also supports that the most stable species formed is ML type for the three metal ions under study (Table 4). Among three metal ions ML type of complex is found to be the most stable for iron metal. Bond lengths, bond angles, total energy, and heat of formation for ML type of complex of the all metal ions are calculated and it is found that the metal complexes possess distorted octahedral geometry (Figures 9(a), 9(b), and 9(c); Table 4).

## 4. Conclusions

The solution studies of trenhynaph by potentiometric and spectrophotometric methods revealed that three protonation constants can be assigned to hydroxyl group of naphthol moiety. The formation constants of its metal complexes with Fe(III), Cr(III), and Al(III) were determined in solution under similar adopted experimental conditions showing the formation of ML as the single major species at physiological pH. The calculated log *β* values are 29.37, 20.94, and 20.49 for Fe(III)-L, Cr(III)-L, and Al(III)-L, respectively. Nine unit higher log *β* values for Fe(III)-L indicate the admirable affinity of the ligand towards Fe(III) than Cr(III) and Al(III), rendering it as more potent iron chelators at physiological pH. Further, the pM values at pH = 7.4 for Fe(III) complex with trenhynaph are found to be 2 to 3 units more as compared to structurally similar chelators, deferiprone (medically applied drug for the iron intoxication) and transferrin (the main Fe binding protein in plasma) indicating higher selectivity of the ligand towards iron in its trivalent state. The high binding affinity of the ligand towards Fe(III) metal ion can be utilized for the removal of Fe(III) in iron overload chelation therapy. Also, the noticeable electronic spectral changes of the complex at pH~7 with the variation in pH support the ligand for potential application as an optical sensor towards Fe(III) metal ion in biological systems. Hence, synthesis of such ligands and their derivatives will open up a perspective towards the development of new optical biosensors.

## Supplementary Material

Supplementary material consists of two figures. Figure 1 represents the 1H NMR spectra of the ligand trenhynaph. It shows peaks at 3.0039 ppm (t, 6H) and 3.7633 ppm (t, 6H) which can be attributed to the methylene protons. The signal obtained at 9.0615 ppm (*d*, 3H) shows the presence of imine proton. The peaks obtained at 6.698 (d, 3H), 7.6177 (d, 3H), 7.547 (d, 3H), 7.1334 (t, 3H), 7.3272 (t, 3H), and 7.9926 (d, 3H) corresponds to the naphthyl protons. A peak at 14.1123 ppm (d, 3H) is also obtained corresponding to the proton of imine nitrogen when ligand exists in keto-imine form. Figure 2 represents the mass spectrum of the ligand. It shows molecular ion peak at  *m*/*z* 609.3, which was expected at 608.27. [M+2] peak is obtained at 610.4.The base peak with 100% intensity is obtained at *m*/*z*  455.2 which corresponds to the fragment C_28_H_30_N_4_O_2 _(454.2). One more important peak at *m*/z 281.1 corresponds to the fragment C_13_H_23_N_3_.

## Figures and Tables

**Table 1:** Experimental and theoretical IR spectral data of ligand.

IR	*ν* (–C=N)	*ν* (–C–O)	*ν* (Ar=C–H)	*δ* (=C–H)	*ν* (–CH_2_)	*ν* (–CH_2_)	*ν* (–C=N)
				(OOP)	Asymmetric	Symmetric	
Experimental (cm^−1^)	1622	1350	3055	764	2940	2821	1622
Theoretical (cm^−1^)	1683	1384	3105	772	2967	2853	1683

**Table 2:** Protonation constants (log⁡⁡K) of the ligand and trenhynaph at *T* = 25 ± 1°C and *µ* = 0.1 M KCl in DMSO/H_2_O (5/95) calculated using Hyperquad 2006 [31].

Equilibrium		log⁡⁡K	
	Assignments	Potentiometry	Spectrophotometry
L + H ⇌ HL	O–H	9.03 ± 0.01	9.04 ± 0.05
HL + H ⇌ H_2_L	O–H	8.02 ± 0.03	8.01 ± 0.06
H_2_L + H⇌H_3_L	O–H	7.12 ± 0.02	7.13 ± 0.09

**Table 3:** Overall (log⁡⁡*β*) for the metal complexes formed by the ligand at *T* = 25 ± 1°C and *µ* = 0.1 M KCl by potentiometry and spectrophotometry.

log⁡⁡*β*		MLH_3_	MLH_2_	MLH	ML	MLH_−1_
Fe(III)	A	39.79 ± 0.02	35.39 ± 0.04	31.91 ± 0.09	29.37 ± 0.01	17.28 ± 0.01
	B	39.78 ± 0.06	35.38 ± 0.07	31.90 ± 0.10	29.36 ± 0.02	17.26 ± 0.09

Cr(III)	A	38.68 ± 0.05	33.54 ± 0.01	26.98 ± 0.09	20.94 ± 0.06	8.54 ± 0.03
	B	38.68 ± 0.08	33.57 ± 0.09	26.94 ± 0.08	20.93 ± 0.08	8.57 ± 0.05

Al(III)	A	34.10 ± 0.07	—	—	20.49 ± 0.06	9.83 ± 0.09
	B	34.11 ± 0.09	—	—	20.47 ± 0.07	9.81 ± 0.08

**Table 4:** The calculated total energies for the various metal complexes formed in solution and structural parameters of M–L type complex through semiempirical PM6 method.

Species	Fe(III)	Cr(III)	Al(III)	Properties			
	*E* (eV)	*E* (eV)	*E *(eV)		Fe(III)–L	Cr(III)–L	Al(III)–L
MLH_3_	−420.4531	−2517.6750	−2419.9876	Bond length (M–N) (A°)	2.133	2.129	2.143
MLH_2_	−1032.6754	−9832.6785	—	Bond length (M–O) (A°)	2.021	2.042	2.078
MLH	−5832.4516	−5130.5612	—	Bond length (M–N) (A°)	2.133	2.129	2.143
ML	−9529.8324	−9246.1048	−8348.1312	Δ*H*_*f*_ (kcal/mol)	−167.43	−142.15	−128.93
MLH_−1_	−890.4563	−720.6745	−960.3451	Bond angle <OMN (°)	91.34	91.99	92.01
