# Supplementary material for: Synthesis of Imine-Naphthol Tripodal Ligand and Study of Its Coordination Behaviour towards Fe(III), Al(III), and Cr(III) Metal Ions
Source: Bioinorg Chem Appl. 2014 Sep 8;2014:915457. doi: 10.1155/2014/915457 (PMC4176642; doi:10.1155/2014/915457)

**Supplementary material for the manuscript entitled**: Synthesis of imine-naphthol tripodal ligand and study of its coordination behaviour towards Fe (III), Al (III) and Cr (III) metal ions

FIGURE1. ^1^H NMR spectra of the ligan trenhynaph


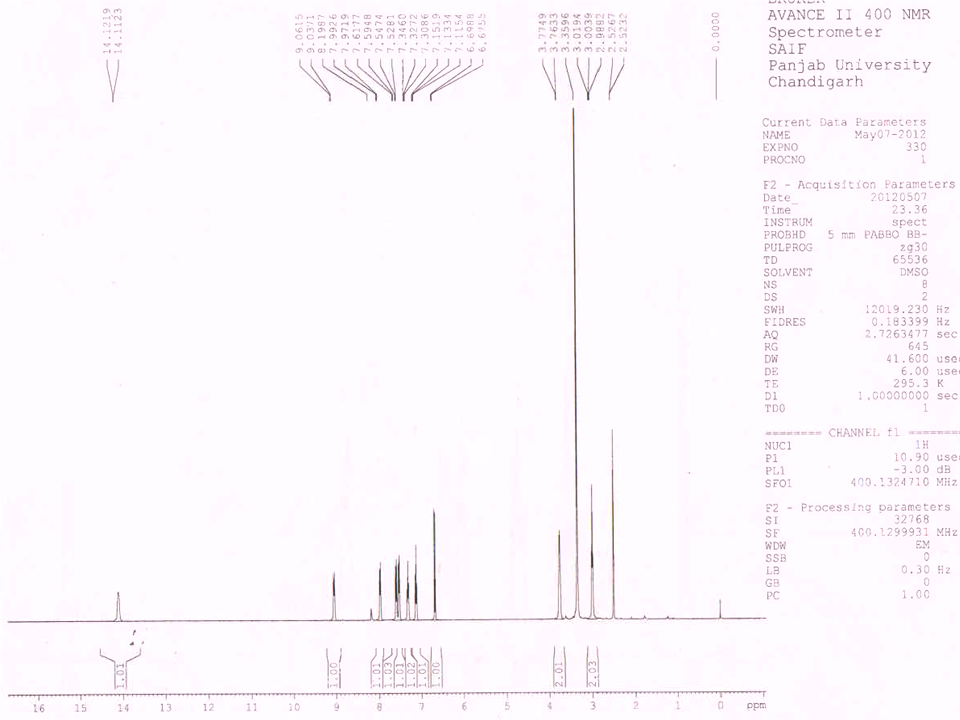


FIGURE 2. Mass spectra of the ligand trenhynaph


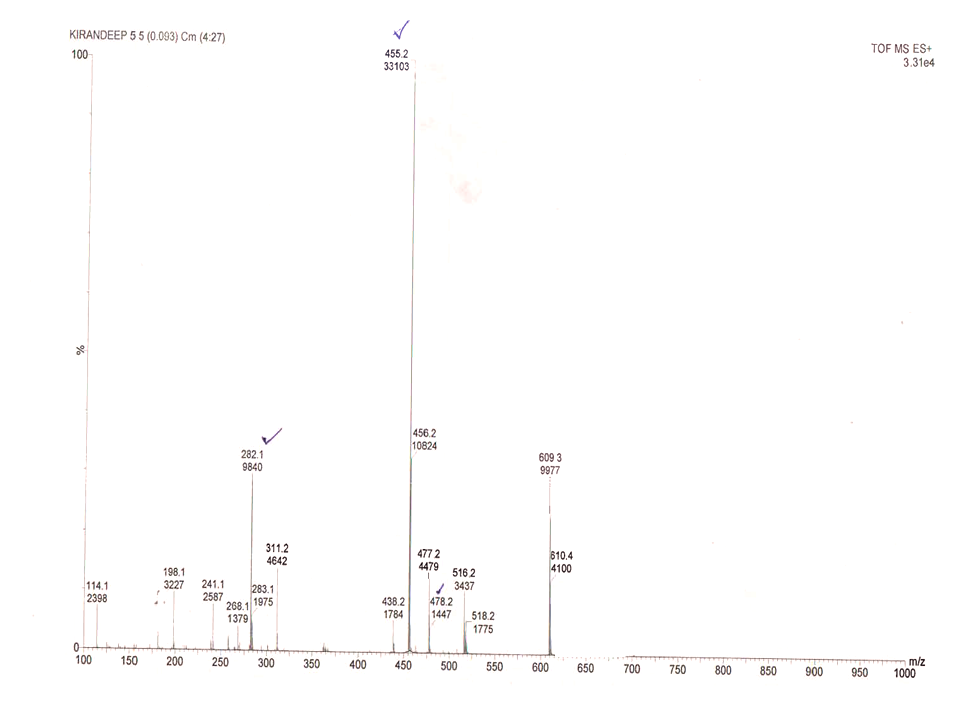

Supplement: Supplementary file 1 — Supplementary material consists of two figures. Figure 1 represents the 1H NMR spectra of the ligand trenhynaph. It shows peaks at 3.0039 ppm (t, 6H) and 3.7633 ppm (t, 6H) which can be attributed to the methylene protons. The signal obtained at 9.0615 ppm (d, 3H) shows the presence of imine proton. The peaks obtained at 6.698 (d, 3H), 7.6177 (d, 3H), 7.547 (d, 3H), 7.1334 (t, 3H), 7.3272 (t, 3H), and 7.9926 (d, 3H) corresponds to the naphthyl protons. A peak at 14.1123 ppm (d, 3H) is also obtained corresponding to the proton of imine nitrogen when ligand exists in keto-imine form. Figure 2 represents the mass spectrum of the ligand. It shows molecular ion peak at m/z 609.3, which was expected at 608.27. [M+2] peak is obtained at 610.4.The base peak with 100% intensity is obtained at m/z 455.2 which corresponds to the fragment C28H30N4O2 (454.2). One more important peak at m/z 281.1 corresponds to the fragment C13H23N3. [file 915457.f1.docx]
